# Elasticity of Cross-Linked Titania Nanocrystal Assemblies Probed by AFM-Bulge Tests

**DOI:** 10.3390/nano9091230

**Published:** 2019-08-29

**Authors:** Andreas Hensel, Clemens J. Schröter, Hendrik Schlicke, Norbert Schulz, Svenja Riekeberg, Hoc Khiem Trieu, Andreas Stierle, Heshmat Noei, Horst Weller, Tobias Vossmeyer

**Affiliations:** 1 Institute of Physical Chemistry, University of Hamburg, Grindelallee 117, D-20146 Hamburg, Germany; 2 Institute of Microsystems Technology, Hamburg University of Technology, Eißendorfer Straße 42, D-21073 Hamburg, Germany; 3 Deutsches Elektronen-Synchrotron (DESY), Notkestraße 85, D-22607 Hamburg, Germany; 4 Physics Department, University of Hamburg, Luruper Chaussee 149, D-22761 Hamburg, Germany

**Keywords:** composite film, bulge test, AFM, titania, nanoparticle, layer-by-layer, attenuation coefficient, elastic modulus, Young’s modulus, XPS

## Abstract

In order to enable advanced technological applications of nanocrystal composites, e.g., as functional coatings and layers in flexible optics and electronics, it is necessary to understand and control their mechanical properties. The objective of this study was to show how the elasticity of such composites depends on the nanocrystals’ dimensionality. To this end, thin films of titania nanodots (TNDs; diameter: ~3–7 nm), nanorods (TNRs; diameter: ~3.4 nm; length: ~29 nm), and nanoplates (TNPs; thickness: ~6 nm; edge length: ~34 nm) were assembled via layer-by-layer spin-coating. 1,12-dodecanedioic acid (12DAC) was added to cross-link the nanocrystals and to enable regular film deposition. The optical attenuation coefficients of the films were determined by ultraviolet/visible (UV/vis) absorbance measurements, revealing much lower values than those known for titania films prepared via chemical vapor deposition (CVD). Scanning electron microscopy (SEM) and transmission electron microscopy (TEM) images showed a homogeneous coverage of the substrates on the µm-scale but a highly disordered arrangement of nanocrystals on the nm-scale. X-ray photoelectron spectroscopy (XPS) analyses confirmed the presence of the 12DAC cross-linker after film fabrication. After transferring the films onto silicon substrates featuring circular apertures (diameter: 32–111 µm), freestanding membranes (thickness: 20–42 nm) were obtained and subjected to atomic force microscopy bulge tests (AFM-bulge tests). These measurements revealed increasing elastic moduli with increasing dimensionality of the nanocrystals, i.e., 2.57 ± 0.18 GPa for the TND films, 5.22 ± 0.39 GPa for the TNR films, and 7.21 ± 1.04 GPa for the TNP films.

## 1. Introduction

Functional materials assembled from inorganic nanocrystals are currently in the focus of intense research efforts aiming at various advanced applications, such as flexible electronics [1,2,3], flexible displays and photovoltaic cells [4,5], chemical and physical sensors [6,7,8], as well as protective and photocatalytic coatings [9]. Obviously, such applications require specific mechanical properties of employed materials. Thus, there is an increasing need for studying the various parameters controlling the mechanical characteristics of thin films fabricated from nanocrystal composites. However, measuring the mechanical properties of these materials accurately is a challenging task.

In previous studies, the mechanical properties of supercrystals from ligand-stabilized inorganic nanoparticles were investigated by nanoindentation experiments. Using this technique, Shevchenko and coworkers [10] and Pileni and coworkers [11,12,13,14,15] showed that the stiffness of nanocrystal assemblies results from the complex interplay of various parameters, including the chemical nature of the nanocrystals, their size and crystallinity, their order and density of packing, the chemical binding between the ligands and the nanocrystal cores, and ligand–ligand interactions. Depending on these parameters, the stiffness of nanocrystal solids was found to vary from a few MPa up to ~7 GPa. In another example, Dreyer et al. [16] employed nanoindentation, uniaxial compression, and microcantilever tests to study the mechanical properties of iron oxide nanoparticle supercrystals. After thermally induced cross-linking of the ligand matrix a very stiff material was obtained with elastic moduli ranging from ~20 to ~80 GPa. Furthermore, freestanding mono- and multilayers of ligand-stabilized nanocrystals have been subjected to atomic force microscopy (AFM) force-deflection measurements to assess their elastic properties. Using this method, He et al. [17] studied freestanding monolayers of Au, Fe/Fe_3_O_4_, and CoO nanocrystals. They reported elastic moduli ranging from 1–14 GPa depending on the type of ligand–nanocrystal and ligand–ligand interactions.

Bulge tests represent an interesting alternative approach to study the mechanical properties of thin films without interferences from an underlying substrate. In such tests, the films are deposited onto substrates with apertures to form freestanding membranes. By applying a well-controlled over- or underpressure the membranes are bulged and the membranes’ elastic properties can be extracted from obtained pressure-deflection data [18]. Usually, the deflection of the membranes is observed either by optical means, e.g., an interferometric setup [19,20,21] or by AFM [22,23,24,25]. Besides being applicable to studying the elastic properties of ultrathin membranes with thicknesses in the range of a few nanometers, the technique also offers another advantage: Because in a bulge experiment the whole membrane is uniformly strained the mechanical properties are sampled over a much larger area than in locally confined indentation or AFM force-deflection experiments. Thus, these measurements are more robust against local inhomogeneity. Additionally, it has been shown that bulge experiments are suited for studying the materials’ viscoelastic creep [24].

Previously, AFM-bulge experiments were used to characterize the mechanical properties of thin films consisting of spherical gold nanoparticles cross-linked by α,ω-alkanedithiols [26]. The focus of the present study is placed on the elastic properties of thin films prepared from differently shaped titania nanocrystals (TNCs). In general, thin films and coatings containing titania nanocrystals are of great interest due to their optical transparency, high refractive index, strong ultraviolet (UV) absorbance, photocatalytic activity, and mechanical reinforcement. Here, the layer-by-layer spin-coating approach [27] was used to prepare thin films of titania nanodots (TNDs), nanorods (TNRs), and nanoplates (TNPs). In order to facilitate regular film deposition, the nanocrystals were deposited alternatingly with 1,12-dodecanedioic acid (12DAC), which was applied to cross-link the nanocrystals. 12DAC was chosen as cross-linker because of its simple molecular structure and due to the high affinity of carboxylic acid groups to titania surfaces. After transferring the films onto substrates with circular apertures they were subjected to AFM-bulge tests. These experiments provide first insights into the mechanical properties (elasticity, pre-stress) of nanocrystal films assembled from differently shaped titania nanocrystals.

## 2. Materials and Methods 

### 2.1. Materials and Chemicals

Silicon wafers (SEMI standard prime; thickness: 300 ± 10 µm; orientation: (100); both sides polished; n-doped, resistivity: 1–10 Ω·cm Silicon Materials, Kaufering, Germany) were used to prepare substrates with circular apertures using deep reactive ion etching (DRIE). A description of this process can be found in the Supplementary Materials (Figures S1 and S2). ACTA AFM cantilevers (k ~40 N·m^−1^, f_0_ ~300 kHz; JPK Instruments, Berlin, Germany) were used for film thickness measurements and NSG01 AFM cantilevers (k ~5.1 N·m^−1^, f_0_ ~150 kHz; NT-MDT Spectrum Instruments, Moscow, Russia) were used for bulge tests. Quartz substrates (GE214, 22 × 22 mm^2^, thickness: 0.5 mm; surface roughness: <0.5 nm) were purchased from Won Ik Quartz Europe GmbH (Geesthacht, Germany).

Titanium(IV)chloride (TiCl_4_, 99%), 1-octadecene (ODE, 90%), oleic acid (OLAC 90%), oleylamine (OLAM, 70%), 1,12-dodecanedioic acid (12DAC, 99%), and 1-butanol (p.a.) were purchased from Sigma Aldrich (Munich, Germany), poly(vinyl alcohol) (PVAl, 30,000 g·mol^−1^, partially hydrolyzed) and triethoxy (isobutyl) silane were purchased from Merck (Darmstadt, Germany). Titanium (IV) fluoride (TiF_4_, 99%) was purchased from Alfa Aesar (Kandel, Germany) and sulfuric acid (96%), hydrogen peroxide (30%), chloroform (p.a.), 2-propanol (p.a.), and tetrahydrofuran (p.a.) were purchased from VWR (Darmstadt, Germany). Unless otherwise stated, all chemicals were used as received without purification. Deionized (DI) water (resistivity 18.2 MΩ·cm) was purified using a Millipore Simplicity system (Merck, Darmstadt Germany). Diethylene glycol stabilized titania nanodots (TNDs, diameter: 7 ± 3 nm) were purchased from CAN GmbH (Hamburg, Germany).

### 2.2. Synthesis of Titania Nanorods (TNRs) and Nanoplates (TNPs)

These nanoparticles were synthesized via a seeded growth process [28] following the method of Gordon et al. [29] with minor modifications. Standard Schlenk-line techniques were used to work under nitrogen atmosphere. For the synthesis of TNRs, a mixture of 120 mL ODE, 7.2 mL OLAC, and 120 mL OLAM were degassed at 120 °C for 1 h. The mixture was allowed to cool down and 5.0 mL precursor solution (0.50 mL (4.6 mmol) TiCl_4_ was dissolved in a degassed mixture of 20 mL ODE and 6.8 mL OLAC) were added at 60 °C under stirring. Under continued stirring the reaction mixture was heated to 290 °C and kept at this temperature for 10 min. By using a syringe pump, 20 mL of the precursor solution were fed continuously over a period of 30 min into the reaction mixture while keeping the temperature at 290 °C. Stirring was then continued at this temperature for 15 min. Afterwards the mixture was allowed to cool down to room temperature and a white precipitate was formed, which was separated from the supernatant by centrifugation (5000× *g*). In order to purify the TNRs they were re-dispersed in ~15 mL chloroform and precipitated again by adding ~15 mL of 2-propanol. After centrifugation the re-dispersion/precipitation procedure was repeated once. Finally, the obtained precipitate was dispersed in 10 mL chloroform to provide the TNR stock solution with a particle concentration of 17.5 mg·mL^−1^. TNPs were prepared following the same procedure. Here, the precursor solution contained 0.56 g (4.6 mmol) TiF_4_ instead of TiCl_4_. The particle concentration of prepared TNP stock solution (in chloroform) was 15.8 mg·mL^−1^. Caution: Hydrofluoric acid (HF) is formed as a side product when using TiF_4_ as precursor. HF is highly hazardous.

### 2.3. Surface Modification of Titania Nanodots (TNDs)

Commercially acquired TNDs were stabilized by diethylene glycol, rendering them dispersible in water. However, for the layer-by-layer preparation of TND films (see below) it was necessary to transfer the particles into chloroform. Thus, the original diethylene glycol ligands were exchanged by OLAM: 1 g of purchased TNDs was mixed with 5 mL OLAM, 15 mL toluene and 5 mL 2-propanol. The resulting mixture was stirred for 7 h until a clear yellowish solution was obtained. The TNDs were then precipitated by the addition of 2-propanol, separated from the supernatant by centrifugation (6000× *g*), and re-dispersed in chloroform. To remove excess ligands the precipitation, centrifugation, and re-dispersion steps were repeated twice. The particle concentration of thus obtained TND stock solution (in chloroform) was 50 mg·mL^−1^. The success of ligand exchange was confirmed by elemental analysis. 

### 2.4. Layer-by-Layer (LbL) Preparation of Titania Nanocrystal (TNC) Films

Quartz substrates were cleaned with concentrated sulfuric acid at 80 °C for 2 h and washed with DI water. The substrates were placed into acetone and treated using an ultrasonic bath (Bandelin Sonorex; BANDELIN, Berlin, Germany) for 15 min, washed with water and dried under nitrogen flow. Using a spin-coater (Süss MircoTec LabSpin6, SÜSS Micro Tec SE, Garching, Germany) a sacrificial polymer layer was deposited by applying 50 µL of a 2.5 wt% solution of PVAl in DI water onto the quartz substrate, which was then rotated at 3000 rpm. Next, 25 µL of 12DAC solution (7.4 mM in 2-propanol) were applied onto the rotating substrate, followed by applications of 25 µL TNC solution (adjusted to ~12.5 mg·mL^−1^ in chloroform) and 2 × 25 µL of the 12DAC solution. After each application a waiting time of 30 s was kept. The alternating deposition of TNCs and the 12DAC was then repeated several times until a TNC film of desired thickness was obtained. The samples were kept overnight in the 12DAC solution to enhance cross-linking of TNCs [27]. Finally, the films were washed with 2-propanol and dried under ambient air.

### 2.5. Preparation of Freestanding TNC Membranes

Freestanding TNC membranes were prepared by transferring respective cross-linked films from their quartz substrates onto silicon substrates featuring circular apertures (60 to 100 µm). To this end, the quartz substrate with the TNC film on top was placed into a glass container and a small amount of DI water was added until the edges of the film were exposed to water but the top surface of the film remained uncovered. Within 1–2 h, the interfacial PVAl layer between the substrate and the TNC film dissolved. Then, the water level was slowly raised by adding more water. The quartz substrate remained on the bottom of the container while the detached TNC film floated freely on the water surface.

Before transferring the TNC film onto a silicon substrate, the latter was cleaned in concentrated sulfuric acid (2 h, 80 °C). Afterwards, it was treated for 2 h at 60 °C with freshly prepared peroxymonosulfuric acid, i.e., a mixture of hydrogen peroxide (30%) and sulfuric acid (96%) 1:1 (*v*/*v*). Caution: Mixing the two components is strongly exothermic and the resulting mixture is extremely oxidizing and can react explosively with organic materials. After washing with DI water and 2-propanol, the substrate was immersed into a solution of triethoxy(isobutyl) silane in 1-butanol (2.5 vol%) for ~12 h. By this treatment the substrate surface turned from hydrophilic to hydrophobic. The substrate was then washed with 2-propanol, baked at 100 °C for 1 h, washed with DI water, and dried under ambient air. Using the surface modified silicon substrate the TNC film was skimmed manually from the water surface and allowed to dry under ambient conditions. 

### 2.6. General Characterization of TNCs and Substrate-Supported TNC Films

The crystal structure of the TNCs was characterized using X-ray powder diffraction (XRD; PANalytical X’PERT Pro diffractometer, Malvern Panalytical, Malvern, UK; copper anode, 45 kV, 40 mA, Bragg-Brentano geometry). Thermogravimetric analysis (TGA) was performed using a Netzsch TGA 209 F1 Iris system (NETZSCH, Selb, Germany). For these measurements the samples (~50 mg) were placed into an alumina crucible, heated to 100 °C, kept at that temperature for 15 min, and then heated under nitrogen flux to 900 °C at a heating rate of 20 °C·min^−1^. The shape and size of the TNCs, and the morphology of cross-linked TNC films were characterized using transmission electron microscopy (TEM; Jeol JEM-1011, Jeol Ltd. Tokyo, Japan, LaB_6_ cathode, 100 kV; Philips CM 300, LaB_6_ cathode, operated at 200 kV). For these measurements the TNCs or TNC films were transferred onto carbon coated copper grids. The sizes and size distributions of TNRs and TNPs were determined by evaluating 200–300 nanocrystals using the software ImageJ. The morphology and thickness of cross-linked TNC films were studied by scanning electron microscopy (SEM; LEO-1550 Gemini, ZEISS, Oberkochen, Germany) and atomic force microscopy (AFM; JPK NanoWizard 3, 100 µm scanner, JPK, Berlin, Germany). All AFM images were acquired in intermittent contact mode. Film thicknesses were determined by measuring step profiles at the edges of the films. AFM data analysis was performed using the Gwyddion V2.41 software package. Optical microscopy (OM) was performed using a Zeiss Axiovert S100 microscope (ZEISS, Oberkochen, Germany) and ultraviolet/visible (UV/vis) absorbance spectra were recoded using a Varian Cary-50 spectrometer (Agilent Technologies, Santa Clara, CA, USA).

X-ray photoelectron spectroscopy (XPS) measurements were carried out using a high-resolution two-dimensional delay line 230 detector [30]. A monochromatic Al Kα X-ray source (photon energy 1486.6 eV; anode operating at 15 kV) was used to provide the incident radiation. XP spectra were recorded in fixed transmission mode. Pass energies of 40 eV and 20 eV were chosen for the survey and region scans, respectively, resulting in an overall energy resolution better than 0.4 eV. Charging effects were compensated by using a flood gun. The binding energies were calibrated based on the graphitic carbon 1s peak at 285.0 eV.

### 2.7. Atomic Force Microscopy Bulge Tests

These measurements were performed using the same methods and, essentially, the same experimental setup as described in our previous study [26]. For conducting an AFM-bulge experiment the silicon substrate carrying the freestanding TNC membrane was fixed onto a custom made sample holder using double-sided sticky tape (TESA 05338, tesa SE, Norderstedt, Germany). The cantilever was positioned close to the substrate’s aperture and a full scan topographic image of the bulge (100 × 100 µm^2^, resolution 512 × 512 px^2^) was acquired whilst applying a constant nitrogen overpressure of ~1 to ~3 kPa (depending on aperture size, film thickness, and stiffness of the membrane) to the bottom side of the freestanding membrane. For acquiring stress-strain data, sets of line scans (0.997 × 100 µm^2^, resolution 5 × 512 px^2^) traversing the apex of the bulge were recorded after stepwise varying the applied pressure within the range of typically 0 to ~6 kPa (depending on aperture size, film thickness, and stiffness of the membrane). The stress–strain data were extracted from AFM data using the circular-fit method, as described in detail previously [26].

All AFM measurements were performed in intermittent contact mode. Usually, a set point of ~70% of the lock-in amplitude was chosen to enable stable imaging conditions. All bulge tests were conducted at room temperature and ambient humidity.

## 3. Results and Discussion

### 3.1. TiO_2_ Nanocrystals (TNCs)—Dots, Rods, and Plates

**Figure 1:**
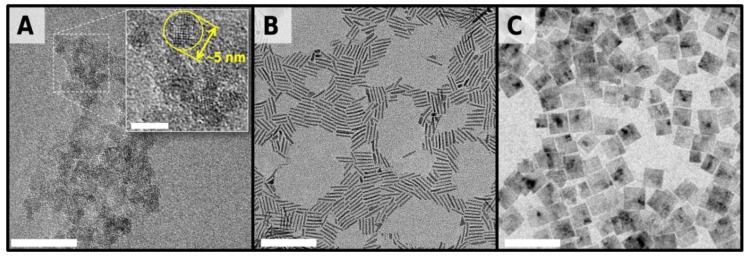
TEM micrographs of synthesized titania nanocrystals (TNCs): (**A**) titania nanodots (TNSs; scale bar: 20 nm, inset: 5 nm), (**B**) titania nanorods (TNRs; scale bar: 100 nm), and (**C**) titania nanoplates (TNPs; scale bar: 100 nm).

Diethylene glycol (DEG) stabilized titania nanodots (TNDs) were acquired commercially as yellowish granular material, which was dispersible in water. For the spin-coating fabrication of TND films (see below) it was necessary to transfer the particles into chloroform. To this end the original DEG ligands were, at least to some extent, exchanged by oleylamine (OLAM). The ligand exchange was confirmed by an elemental analysis (Supplementary Materials, Tables S1 and S2), revealing an N/C ratio of ~1/21. This finding suggests a ~1.6 fold molar excess of OLAM over residual DEG. Using thermogravimetric analysis (TGA) a total organic mass fraction of ~40% was measured (Supplementary Materials, Figure S3). Assuming a 0.2 nm^2^ molecular footprint of OLAM [31] and a spherical particle shape with a diameter of 5 nm, the total amount of OLAM corresponded roughly to a monolayer coverage of the TNDs’ surface (Supplementary Materials, Figure S4). However, a precise determination of the particle size via transmission electron microscopy (TEM) was difficult because the TNDs aggregated on the TEM substrates and occasionally observed isolated nanocrystals showed only faint imaging contrast. The crystalline domains, as seen in the TEM image in Figure 1A, indicated particle diameters roughly in the range from 3 to 7 nm, which is in agreement with the specifications provided by the manufacturer (7 ± 3 nm). Powder XRD measurements were consistent with the anatase polymorph (Supplementary Materials, Figure S5). The crystalline domain sizes were determined using the widths of the (101) and (200) diffraction peaks according to the Scherrer equation [32]. In agreement with the TEM analysis this method provided crystalline domain sizes of 5–6 nm (Table S4).

Titania nanorods (TNRs) were synthesized following the seeded growth approach of Gordon et al. [29]. As the procedure requires the addition of OLAC as oxygen source and an excess of OLAM as shape-directing agent, this method provided TNRs, which were stabilized by a mixture of OLAM and OLAC. Elemental analysis revealed an N/C ratio of ~1/32, suggesting an OLAM/OLAC molar ratio of ~1.3/1.0 (Supplementary Materials, Table S3). As determined by TGA, the total organic mass fraction was ~25%. Assuming a footprint of 0.2 nm^2^ for both, OLAM [30] and OLAC [33], suggests that the amount of ligands within the final TNR stock solution corresponded to roughly half a monolayer coverage of the nanorods’ surface (Supplementary Materials, Figure S4). TEM characterization revealed an average rod length of 29.3 ± 3.7 nm with an average diameter of 3.4 ± 0.4 nm. A representative TEM image is shown in Figure 1B. Powder XRD measurements revealed that brookite was the dominant crystalline phase, while a small amount of anatase phase was still present, in agreement with literature [28,29] (Supplementary Materials, Figure S5).

Titania nanoplates (TNPs) were synthesized using the same approach as for the TNR synthesis, with minor modifications [29]. Here, titanium(IV)tetrafluoride was used as a precursor. Since fluoride ions bind strongly to the {001} facets of the anatase polymorph the crystal growth in these directions is inhibited. Thus, resulting particles have a plate-shaped morphology with a quadratic base area. TEM analysis revealed an average edge length of 33.6 ± 4.2 nm and a plate thickness of ~6 nm. A representative TEM image is shown in Figure 1C. Powder XRD measurements confirmed the anatase phase (Supplementary Materials, Figure S5). As in the case of the TNR synthesis, OLAC was used as oxygen source and a huge excess of OLAM was added to the reaction mixture as an additional shape-directing agent. Accordingly, the synthesis provided OLAM/OLAC-stabilized TNPs. Elemental analysis revealed an N/C ratio of 1/25, suggesting an OLAM/OLAC molar ratio of ~2.7/1.0 (Supplementary Materials, Table S3). As determined by TGA, the total organic mass fraction was ~25%, suggesting that the amount of OLAM/OLAC ligands within the final TNP stock solution corresponded to roughly a monolayer coverage of the nanoplates’ surface (Supplementary Materials, Figure S4).

The properties of the three TNC samples used for film preparations are summarized in the Supplementary Materials (Table S5).

### 3.2. Layer-by-Layer (LbL) Preparation and Characterization of Substrate Supported TNC Films

**Scheme 1:**
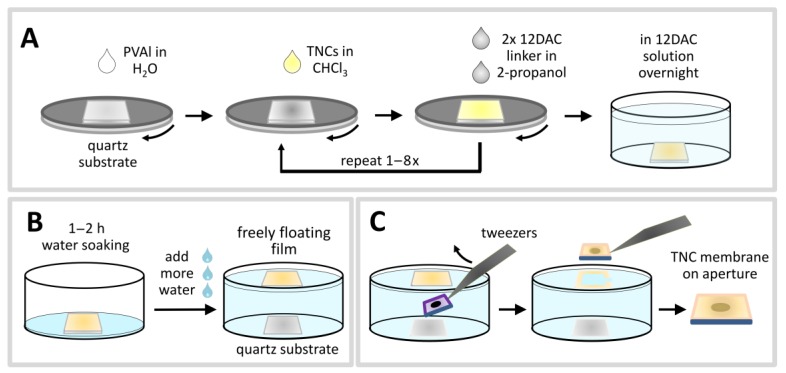
(**A**) Layer-by-layer (LbL)-fabrication of TNC films via spin-coating: A quartz substrate is coated with a sacrificial polyvinyl alcohol (PVAl) layer. The TNCs and 12DAC are deposited repeatedly onto this layer. Finally, the TNC film is immersed into 12DAC solution overnight to cross-link the TNCs. (**B**) Detachment of TNC films from quartz substrates: The coated substrate is placed into a container and a small volume of water is added to dissolve the sacrificial PVA layer. By slowly raising the water level the TNC film is detached from the substrate and floats on the water surface. (**C**) Preparation of freestanding TNC membranes: The TNC film is manually skimmed off the water surface using a hydrophobic silicon substrate with a circular aperture.

**Figure 2:**
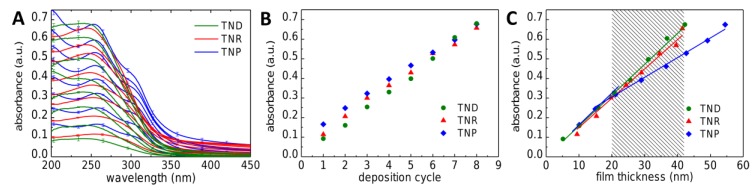
(**A**) UV/vis-absorbance spectra of TND, TNR, and TNP films measured after each cycle (1–8) of the LbL spin-coating deposition. PVAl-coated quartz slides were used as substrates. (**B**) Absorbance at 250 nm of TND, TNR, and TNP films vs. number of deposition cycles. (**C**) Absorbance at 250 nm of TND, TNR, and TNP films vs. film thickness. The shaded area indicates the range of thicknesses of the films studied by atomic force microscopy bulge tests (AFM-bulge tests).

For the layer-by-layer (LbL) preparation of TNC films freshly cleaned quartz substrates were first spin-coated with polyvinyl alcohol (PVAl). The water-soluble polymer formed a smooth homogeneous layer with a thickness of 100 to 200 nm. This film served as sacrificial layer to enable the preparation of freestanding TNC membranes, as detailed below (Scheme 1). 1,12-dodecanedioic acid (12DAC) and the TNCs were then deposited alternatingly via spin-coating onto the PVAl layer. Scheme 1A depicts the whole spin-coating process. Representative absorbance spectra of TND, TNR, and TNP films recorded after each completed deposition cycle are shown in Figure 2A. The TND and TNR films showed a broad absorbance band below ~350 nm, with a maximum developing at ~250 nm for the thicker TNR films. Similar absorbance spectra were reported for thin titania films prepared by a sol–gel process [34]. The TNP films featured a more structured absorbance spectrum with a maximum at ~255 nm and a pronounced shoulder at ~305 nm, similar to thin TiO_2_ films prepared via chemical vapor deposition (CVD) [35]. Plotting the absorbance at 250 nm against the number of deposition cycles (Figure 2B) confirmed the well-controlled increase in film thickness with each deposition cycle. The actual thickness of the TNC films was determined by AFM after transferring them onto silicon substrates (see below). Figure 2C shows the absorbance (at 250 nm) plotted vs. film thickness. Obeying Lambert’s law the absorbance of the TND and TNR films increased linearly with increasing thickness, revealing that the optical density did not change with the number of deposition cycles. The linear fits to the data returned decadic attenuation coefficients of 1.58 × 10^5^ cm^−1^ and 1.50 × 10^5^ cm^−1^ for the TND and TNR films, respectively. In case of the TNP film, the absorbance increased more steeply with the first two deposition cycles (decadic attenuation coefficient: 1.63 × 10^5^ cm^−1^) and then continued to increase linearly with a significantly smaller slope (1.00 × 10^5^ cm^−1^). This finding suggests that the volume fraction of titania was significantly lower within the upper layers than within the first two layers deposited. Furthermore, comparing the attenuation coefficients to those of the other two TNC films suggests that the volume fraction of titania within the upper layers of the TNP film was significantly lower than that of the TND or TNR films (provided that the differently shaped titania nanocrystals had similar volume-normalized extinction coefficients). This finding suggests that during the first two deposition cycles the nanoplates aligned with the substrate’s surface and, therefore, formed a quite densely packed arrangement. With increasing thickness, however, the film surface became rougher and TNPs were deposited in a more randomly packed arrangement with a more porous morphology.

For comparison, Mills et al. [35] studied the optical properties of TiO_2_ thin films deposited via CVD onto quartz substrates. They measured a decadic attenuation coefficient of ~2.20 × 10^5^ cm^−1^ (at 250 nm). Assuming similar volume-normalized extinction coefficients for the differently shaped nanocrystals, this comparison suggests that all three TNC films had a lower volume fraction of titania than CVD deposited films, most likely due to the presence of organic material (ligands and the 12DAC linker) and a more porous structure (see structural characterization below).

For successfully performing the LbL deposition of TNC films it was mandatory to use orthogonal solvents. The TNCs were dissolved in chloroform whereas 12DAC was dissolved in 2-propanol before spin-coating. These solvents do not dissolve the sacrificial PVAl layer. Furthermore, 2-propanol does not dissolve the hydrophobic OLAM/OLAC stabilized TNCs and, thus, the TNCs are not washed off the substrate when applying the 12DAC solution. After the final spin-coating cycle, the coated substrates were immersed overnight into the 12DAC solution to enhance cross-linking of TNCs. Finally, excess 12DAC was removed by washing the films with 2-propanol. When treating the films with chloroform the obtained coatings did not re-dissolve or degrade, suggesting that the particles were successfully cross-linked. Since carboxylic acids are known to bind strongly to TiO_2_ surfaces, it is expected that OLAM ligands at the TNC surfaces are replaced, at least to some extent, by 12DAC, leading to a cross-linked TNC network.

In order to further characterize the TNC films they were transferred onto suitable substrates. To this end, the TNC coated quartz substrates were placed into a glass container, which was slowly filled with water to dissolve the sacrificial layer of PVAl underneath the TNC film (Scheme 1B). This way, the TNC films detached from their original substrates and remained freely floating on the water surface. The films were then skimmed manually from the water surface using suitable substrates.

The thickness and surface roughness of the TNC films were measured by AFM (Supplementary Materials, Figures S6 and S7). For this purpose, the films were transferred onto silicon substrates. Measured film thicknesses are displayed in Figure 2C and correlated with the UV-absorbance at 250 nm. Typical arithmetic average surface roughnesses (*R*_a_) of TNC films with thicknesses of 20 to 25 nm were 4.17 nm, 1.58 nm, and 4.56 nm for the TND, TNR, and TNP films, respectively.

**Figure 3:**
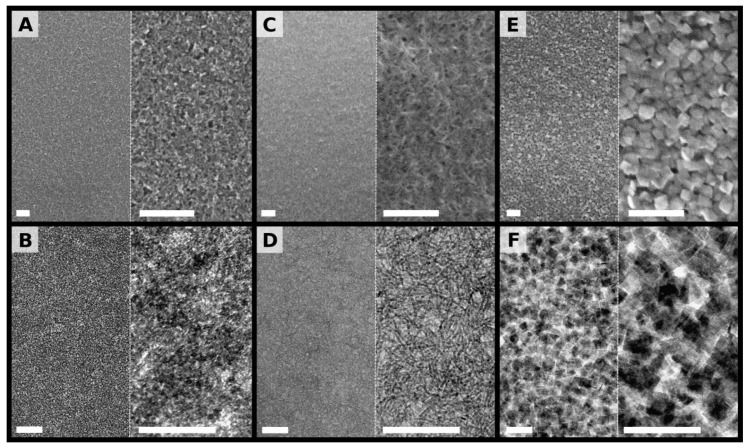
SEM (**A**,**C**,**E**) and TEM (**B**,**D**,**F**) micrographs of TNC films. In each panel two micrographs at different magnifications are shown. (**A**) and (**B**) show TND films obtained after four deposition cycles; (**C**) and (**D**) show TNR films obtained after four and three deposition cycles, respectively; (**E**) and (**F**) show TNP films obtained after five deposition cycles, respectively. Scale bars in (**A**), (**C**), and (**E**): 200 nm; scale bars in (**B**), (**D**), and (**F**): 100 nm.

Figure 3 shows SEM and TEM images of the three TNC films transferred onto silicon substrates and carbon coated copper grids, respectively. At low magnification the TND films show a quite homogeneous coverage of the substrates (Figure 3A, left). At higher magnification, however, a disordered microporous structure is observed (Figure 3A, right). The TEM images shown in Figure 3B confirm the formation of a granular film, which forms a quite uniform layer on the µm-scale, but appears disordered and rather inhomogeneous on the ~100 nm scale. This finding is consistent with relatively high surface roughness determined by AFM.

The low magnification SEM image of the TNR film (Figure 3C, left) reveals a uniform and homogeneous coverage of the substrate, similar as the TND film. In contrast, however, a fibrous porous structure is clearly seen, especially at higher magnification (Figure 3C, right). This fibrous structure is also observed in the TEM images shown in Figure 3D. The TEM images indicate that the TNRs do not have any preferred orientation within the xy-plane of the film. Further, there is no indication of extended side-by-side alignment and bundling.

Due to the larger size of the TNPs, respective films have a more coarse-grained structure, as seen in the SEM images in Figure 3E. Here, single particles can easily be recognized. On the µm-scale the film forms a uniform layer covering the substrate. At higher magnification (Figure 3E, right panel) a highly disordered, porous structure with randomly orientated TNPs is observed. This finding is in agreement with the relatively high surface roughness determined by AFM (see above). The TEM images shown in Figure 3F confirm the random orientation of the TNPs. There is no obvious indication for alignment or stacking of TNPs.

**Figure 4:**
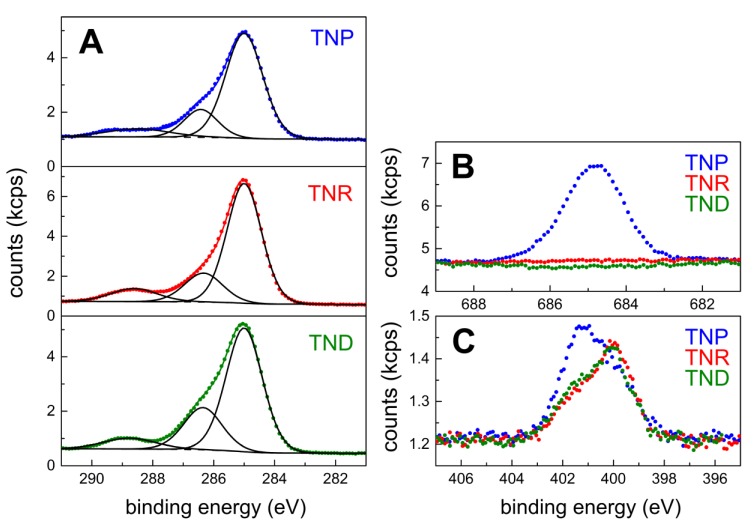
X-ray photoelectron spectroscopy (XPS) data of TNC films. Figure part (**A**) shows the C 1s signals of TNP, TNR, and TND films. The peaks at binding energies around 288.6 and 286.3 eV are attributed to carboxylic acid groups while the peak at around 285.0 eV is attributed to aliphatic carbon. Figure part (**B**) shows the F 1s energy region. Figure part (**C**) shows the N 1s signals of all three TNC films. In (**B**) and (**C**) the baseline level of the TNR and TND film was adjusted to the baseline level of the TNP film for clarity of data presentation.

The chemical composition of the TNC films was analyzed by X-ray photoelectron spectroscopy (XPS). The signals of the C 1s core level confirmed the presence of aliphatic hydrocarbon and carboxy species. The latter are attributed to the carboxylic acid groups of residual OLAC (in the case of TNR and TNP films) and added 12DAC cross-linker, see Figure 4A. A comparison of the XPS data with the findings of the elemental analyses of the TNC samples used for film preparation (see above) reveals that the amount of carboxylic carbon relative to total carbon increased significantly (Supplementary Materials). Thus, these data confirm the deposition of the 12DAC cross-linker during film formation. The assignment of the carboxylic group in the C 1s region at 288.6 eV and 286.3 eV correspond to the O 1s level signals at 532.4 eV and 531.0 eV (Supplementary Materials, Figure S8C).

As expected, the TNP sample contained significant amounts of fluorine from the TiF_4_ precursor, see Figure 4B. Fluoride is known to bind to anatase {001} facets [29]. In the TNR and TND films fluorine could not be detected. In the case of the TNR sample, only a very faint Cl 2p signal (noise level) from the TiCl_4_ precursor could be observed (Supplementary Materials, Figure S8D). 

As seen in Figure 4C, the N 1s signal revealed the presence of at least two nitrogen species. In a previous study [36], the signals at 399.9 eV and 401.5 eV were assigned to free and H-bonded amines, respectively. Thus, these signals may indicate the presence of residual OLAM. However, the assignment of the binding energies of N-containing species on titania surfaces is a matter of debate. For example, a signal at 399.6 eV has also been assigned to O–Ti–N linkages [37] Furthermore, as noted previously, contamination of titania surfaces by N-containing species is unavoidable [36]. Thus, a more specific analysis of the measured N 1s signal is difficult and beyond the scope of our current study.

The XPS survey spectra of the three TNC films as well as and the spectra for the Ti 2p are included in the Supplementary Materials, Figure S8. 

### 3.3. Freestanding TNC Membranes and AFM-Bulge Tests

Freestanding TNC membranes were prepared by skimming the films from the water surface using silicon substrates with circular apertures (Scheme 1C). The diameters of the apertures ranged from 32–111 µm. In initial experiments it was observed that most of the transferred TNC films were dragged into the orifice, stretched, and cracked while drying under ambient air. This failure was attributed to capillary action. Thus, in order to reduce the capillary force the hydrophilic character of the silicon substrates was decreased by treating them with triethoxy(isobutyl)silane. With this modification the majority (~75%) of the TNC films formed stable, freestanding membranes spanning the substrates’ aperture.

**Figure 5:**
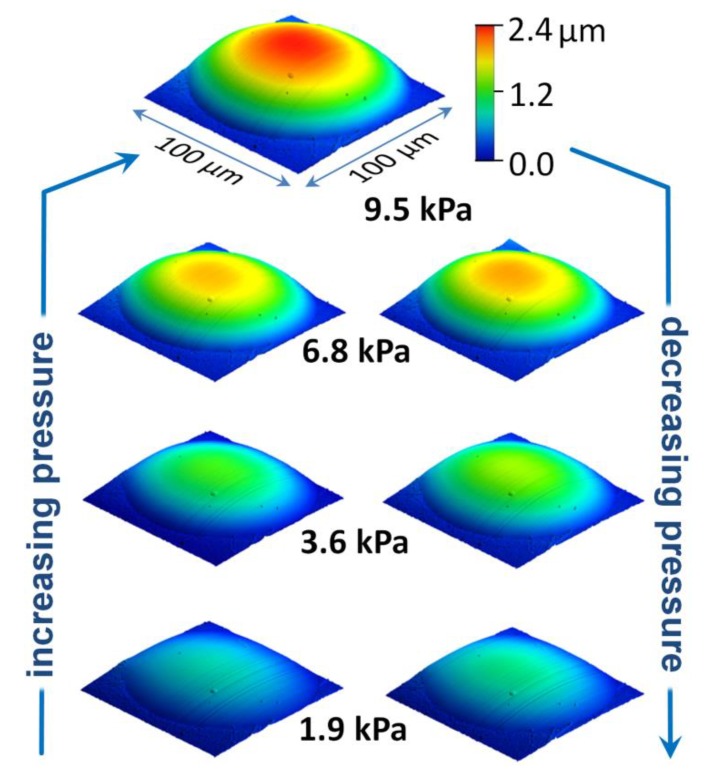
AFM images of a TNR membrane acquired whilst applying constant pressure differences, as indicated. A regular dome was formed above the circular aperture of the substrate. The pressure load was first increased and then decreased to test reversibility of bulge formation.

AFM-bulge tests were performed using the same equipment as in our previous work [26]. In a first preliminary set of experiments, full-scan images (100 × 100 µm^2^, 512 × 512 pixel^2^) of the bulged TNC membranes were acquired whilst applying constant pressure differences ranging from 1 to 10 kPa. At sufficiently high overpressure a regular dome with a circular base area was formed. These images enabled a qualitative assessment of the membranes’ bulging behavior. For example, the series of images in Figure 5 shows the reversible deflection of a TNR membrane under various pressure loads. Similar results were obtained for the TND and TNP membranes. These measurements clearly demonstrate that LbL prepared TNC films are well suited for probing their micromechanical properties via AFM-bulge tests: First, their deflection under pressure load can be controlled and measured accurately with the AFM setup. Second, under pressure load the membranes formed fairly regular bulges that can be analyzed using the spherical cap model with approximations of a thin-walled pressure vessel (see below). Third, during the AFM measurements the membranes remained fixed to the substrate along the aperture’s circumference without indications of detachment.

**Figure 6:**
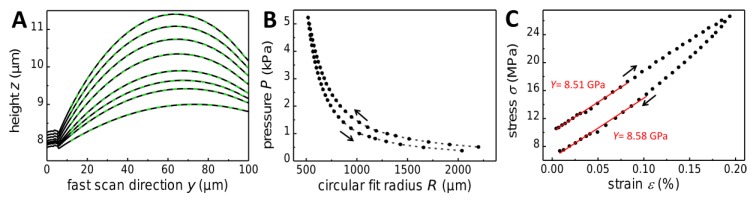
(**A**) AFM scans showing height profiles of a bulged TNR membrane (Supplementary Materials, Table S6: TNR5). The applied overpressures ranged from 0.375 to 5.23 kPa. Dashed green lines are segments of circular fits to the data used to determine the radius *R*. (**B**) Correlation of overpressure *P* and the circular fit radius *R*. During the course of the experiment the pressure was first increased and then decreased (see arrows). (**C**) Stress–strain curves with arrows indicating the loading and unloading directions of the experiment. The slopes of the linear fits to the data at lower strain are 8.51 GPa and 8.58 GPa for loading and unloading, respectively.

To characterize the membranes’ elastic properties quantitatively a full-scan AFM image of the bulge was acquired. This image was used to localize the apex of the bulge. The pressure dependent deflection of the membrane was then measured by acquiring line scans traversing the apex of the bulge. In these experiments the pressure *P* was varied stepwise, typically within the range of ~0.1 to ~6 kPa. A representative set of line scans used to extract the membranes’ biaxial modulus is shown in Figure 6A. According to the model of the thin-walled pressure vessel the biaxial stress *σ* of the bulged membrane is given by Equation (1):

(1) $$\sigma =\frac{PR}{2t}.$$

**Scheme 2:**
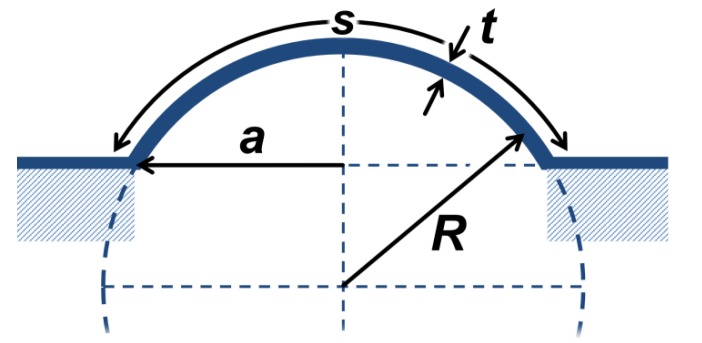
Cross-sectional schematic of the bulged membrane according to the spherical cap model. The membrane’s dome formed above the substrate’s aperture with radius *a* is approximated by a sphere with radius *R*. *t* is the thickness of the bulged membrane and *s* is the arc length traversing the apex of the bulge.

Here, *R* is the radius of the pressure vessel, and *t* is the thickness of the membrane, as illustrated in Scheme 2. This model is applicable as long as *R* >> *t*. The thickness *t* of the membranes was determined by UV/vis spectroscopy, using the calibration curves in Figure 2C. The bulge radius *R* was determined by fitting circles to the measured arc profiles, as indicated in Figure 6A (dashed green lines). This method, which was referred to as “circular-fit method” in a previous study [26], is fairly robust against substrate tilt. Figure 6B shows the change of the bulge radius *R* with increasing and decreasing overpressure *P*.

The strain *ε* of the bulged membranes was determined using Equation (2):

(2) $$\epsilon =\frac{s}{2a}-1.$$

Here, *s* is the arc length of the bulge, which is confined by the aperture’s diameter 2*a* (Scheme 2). Using the bulge radius *R* and the aperture radius *a* the arc length *s* can be computed, according to Equation (3):

(3) $$s=2R\;\arcsin\left(\frac{a}{R}\right).$$

According to Equation (4) the biaxial modulus *Y* and the pre-stress $\sigma_{0}$ can be extracted as the slope and the ordinate intercept of linear fits to the stress–strain data:

(4) $$\sigma =Y\cdot \epsilon +\sigma_{0}.$$

Figure 6C shows the stress–strain correlation diagram of a TNR membrane. The observed deviation of ascending data (loading) from descending data (unloading), which is also seen in the *P* vs. *R* plot (Figure 6B), is due to viscoelastic deformation. As a result, the residual stress of the membrane (i.e., the stress observed at the end of the experiment at zero strain) was somewhat lower than the initial pre-stress $\sigma_{0}$ (which is determined by extrapolating the initial loading curve to the ordinate intercept). This observation is attributed to pure viscous creep and/or retarded elastic deformation, as suggested previously for membranes of alkanedithiol cross-linked gold nanoparticles [26]. Within the lower strain range, both, loading and unloading curves were linear with very similar slopes. Therefore, in the following, only the slopes of linear fits to the stress–strain loading curves were considered to determine the biaxial moduli of the membranes. 

**Figure 7:**
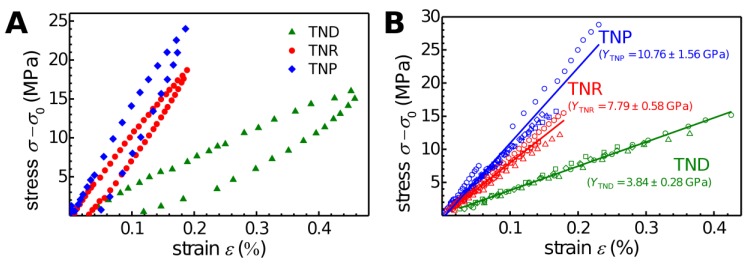
(**A**) Stress–strain data of TND1, TNR5, and TNP3 membranes. (**B**) Stress strain data of all membranes studied. The slope of each solid line represents the averaged biaxial modulus *Y* of the respective membrane material. The biaxial moduli of all individual membranes are provided in the Supplementary Materials (Table S6).

In Figure 7A representative loading and unloading stress–strain data were plotted for the three types of TNC membranes studied. All three materials showed qualitatively similar stress–strain curves. However, the different slopes of these data clearly suggest a decrease of the membranes’ biaxial modulus in the order TNP > TNR >> TND. 

In order to confirm this trend, several membranes of the different TNC materials were prepared and characterized. The stress–strain loading data of all 18 membranes studied are provided in the Supplementary Materials (Figures S9–S11), together with values of their biaxial modulus *Y* and their pre-stress $\sigma_{0}$ (Supplementary Materials, Table S6). Most membranes studied were taut and had positive pre-stress values, scattering between 0.9 to 21 MPa without a clear difference between the three types of membrane materials. Only one TNP membrane was slack and had a negative pre-stress of −8.2 MPa. The scattering of the $\sigma_{0}$ values is most likely due to the manual procedure used to transfer the membranes onto the AFM substrates.

Figure 7B shows the (*σ*–$\sigma_{0}$) vs. *ε* data of all TNC membranes studied. These data clearly confirm that the biaxial modulus decreased in the order TNP > TNR >> TND. Taking into account that for these bulge tests membranes with different thickness, diameter, and pre-stress were used (Supplementary Materials, Table S6), the similarity of extracted biaxial moduli referring to the same type of material was quite remarkable and underlined the robustness of the AFM-bulge test method. The average biaxial modulus *Y* of each membrane material was determined by averaging the slopes of the linear fits to the data for each individual membrane of respective material. In addition, the biaxial modulus was converted into Young’s modulus *E*, according to Equation (5):

(5) $$Y=\frac{E}{1-\nu }.$$

Here, *ν* is the Poisson ratio, which was assumed to equal 0.33 [10,17]. The average values of *Y* and *E* are presented in Table 1 for all three membrane materials.

The mechanical properties of composites from ligand stabilized inorganic nanoparticles depend on various interdependent parameters, including the particles’ size, shape, their spatial arrangement, the size and molecular structure of the ligands and added cross-linkers, the efficiency of ligand–ligand interactions and cross-linking, the chemical binding of the ligands, and cross-linkers to the nanoparticles’ surfaces, which may involve different crystal facets and different crystallite phases, and, in general, the overall volume fraction of stiff (inorganic) to soft (organic) matter, and the materials’ microstructure (e.g., porosity). Here, an increasing stiffness of the TNC films was observed when increasing the TNCs’ size and dimensionality from dots (0D) to rods (1D) to plates (2D). As suggested by the optical attenuation coefficients, the titania volume fractions of the TND and TNR films were similar, nevertheless the elastic modulus of the TNR films was approximately twice as high as that of the TND films. According to TEM and SEM characterization (Figure 3), the higher stiffness of the TNR membranes is attributed to the haystack-like network of stiff TNRs with numerous inter-rod contacts cross-linked by 12DAC. The higher surface roughness of the TND films, as detected by AFM (see above), suggests also that their stiffness was reduced by the more discontinuous film morphology. When further increasing the size and dimensionality of the TNCs by using TNPs for film assembly the stiffness of the composite increased only moderately. SEM and TEM images revealed that the nanoplates were randomly orientated within the TNP films (Figure 3E,F). Accordingly, a coarse-grained porous structure was formed and cross-linking of TNPs via the comparably small 12DAC molecules (~1 nm in length) can occur only at points of close inter-plate contacts. Therefore, the density of cross-linked inter-particle contacts was probably much lower than in the case of TND or TNR films. Thus, due to the larger size of the TNPs (~100× and ~25× larger volume than that of TNDs and TNRs, respectively) these films showed an overall increase in stiffness. However, the particle size effect was most likely compromised by less efficient cross-linking and a discontinuous film morphology. Further, cross-linking of the TNPs may have been compromised additionally by fluoride ions inserted into the {001} surfaces as well as residual OLAC/OLAM ligands, which may have hindered the efficient binding of 12DAC to the nanoparticle surface.

The elastic moduli of our disordered TNC membranes are, in general, similar to those reported for membranes of highly ordered nanocrystals with interdigitating ligand shells, as well as for membranes of gold nanocrystals covalently cross-linked by *α*,*ω*-alkanedithiols [15,17,26] He et al. [17] studied freestanding membranes of Fe/Fe_2_O_3_, Au, and CoO nanocrystals with core diameters ranging from 5 to 14 nm. Due to efficient ligand interdigitation remarkably stiff materials were formed with average elastic moduli ranging from 1 to 14 GPa, as determined by AFM force-indentation measurements. Similar elastic properties were reported by Shevchenko and co-workers [10] and Pileni and coworkers [11,12,13,14,15] who studied the micromechanical properties of supercrystals from densely packed nanocrystals by nanoindentation. These studies showed that the interplay of the nanocrystals’ packing density, the degree of ordering, and the efficiency of ligand interdigitation is crucial for setting the overall material’s mechanical properties. 

According to previous findings, it should be possible to increase the stiffness of TNC nanocomposites by increasing the degree of cross-linking. Liaqat et al. prepared multilayered titania nanoparticle/DOPA-polymer composites [38]. These films, which were prepared in a layer-by-layer spin-coating procedure, consisted of several bilayers, each formed by one ~46 nm thick TND layer (TND diameter: ~8 nm) and one ~4 nm thick polymer layer. After each spin-coating cycle the layers were heated to 120 °C. Since the polymer infiltrated into the nanoparticle layers a strongly cross-linked hybrid network was formed, with strong binding of the polymer’s catechol groups to the nanoparticles’ surfaces. As a result, the composite had a high elastic modulus of 17.5 ± 2.5 GPa, as determined by nanoindentation measurements.

Finally, it is interesting to compare the elastic properties of our TNC membranes with those of titania films prepared by various chemical and physical deposition techniques. For example, Roy et al. [39] prepared nanocrystalline titania films from aqueous solution via hydrolysis of TiCl_4_. Depending on the reaction conditions they obtained porous or dense films with rutile or anatase as dominating phase. Dynamic nanoindentation measurements revealed Young’s moduli of ~13.3 and ~24.5 GPa, respectively. These values are roughly one order of magnitude lower than Young’s moduli of crystalline titania layers deposited onto glass and silicon substrates by reactive pulse magnetron sputtering (170 GPa and 260 GPa for anatase and rutile, respectively) [40]. Gaillard et al. [41] studied titania films with different columnar microstructures prepared via physical vapor deposition (PVD). They showed that the elastic modulus decreased from 87.5 GPa to 16.5 GPa with increasing porosity and tilt angle of the columnar structure. 

## 4. Summary and Conclusions

Thin films of titania nanodots (TNDs; diameter: ~3–7 nm), nanorods (TNRs; diameter: ~3.4 nm; length: ~29 nm), and nanoplates (TNPs; thickness: ~6 nm; edge length: ~34 nm) were prepared via layer-by-layer spin-coating at room temperature. In this process, 12DAC was added to support the film deposition via cross-linking of nanocrystals. A well-controlled layer-by-layer film deposition was confirmed by the regular increase in UV/vis absorbance after each deposition cycle. SEM and TEM images of 20–42 nm thick films revealed a quite homogenous coverage of the substrates on the µm-scale. At higher magnifications a highly disordered arrangement of the nanocrystals was observed. After transferring the films onto substrates featuring circular apertures their elastic properties were evaluated via AFM-bulge tests, revealing average elastic moduli of 2.57 ± 0.18 GPa for TND membranes, 5.22 ± 0.39 GPa for TNR membranes, and 7.21 ± 1.04 GPa for TNP membranes. Compared to the TND membranes, the higher stiffness of the TNR membranes was attributed to the haystack-like structure of cross-linked nanorods and the more continuous film morphology. The increase in stiffness observed for the TNP membranes (compared to the TNR membranes) was attributed to the increased overall nanocrystal size, accompanied by less efficient cross-linking due to the coarse-grained porous morphology with a reduced number of contact points between the randomly oriented TNPs. 

In general, the observed elastic moduli are comparable to those previously reported for highly ordered membranes and supercrystals of nanoparticles with non-covalent ligand–ligand interactions (1–14 GPa) [10,11,12,13,14,15,17]. However, they are lower than those observed for highly cross-linked titania nanoparticle/polymer composites (17.5 ± 2.5 GPa) [38] or superlattices of ligand-stabilized iron oxide nanoparticles with interdigitated and covalently cross-linked ligand shells (~81 GPa) [16]. Based on these findings, our future work is aiming at tuning the micromechanical properties of TNC composites by systematically varying the degree of cross-linking, e.g., by using differently sized, multifunctional cross-linker compounds and by employing different types of chemical interactions for cross-linking and for anchoring the cross-linkers to the nanoparticles’ surfaces. Further future work should address the influence of nanocrystal packing on the mechanical properties. While the films prepared in this study via spin-coating were highly disordered, more ordered and densely packed nanocrystal films should be achievable by using other deposition techniques, such as iterative interfacial nanocrystal assembly, including Langmuir-Blodgett techniques.

## Figures and Tables

**Table 1:** Average biaxial moduli *Y* and elastic moduli *E* of the three different types of TNC membranes.

Material	TND	TNR	TNP
Biaxial Modulus *Y* (GPa)	3.84 ± 0.28	7.79 ± 0.58	10.76 ± 1.56
Elastic Modulus *E* (GPa)	2.57 ± 0.18	5.22 ± 0.39	7.21 ± 1.04

## Supplementary Materials

The following are available online at https://www.mdpi.com/2079-4991/9/9/1230/s1, Figure S1: Deep reactive ion etching process, Figure S2: Scanning electron microscopy (SEM)-Image of a circular aperture, Table S1: Elemental analyses for carbon (C), hydrogen (H) and nitrogen (N), Table S2: Elemental analyses of TNDs, TNRs, and TNPs, Table S3: Molar ratios of the two ligands, Figure S3: Thermogravimetric analyses, Figure S4: Geometries and dimensions, Figure S5: X-ray powder diffraction (XRD) data, Table S4: Evaluation of XRD data, Table S5: Properties of nanocrystal samples used for film preparation, Figure S6: Atomic force microscopy (AFM) images, Figure S7: Height profiles, Figure S8: X ray photoelectron spectroscopy (XPS) Data of TNC films, Figure S9: Stress-strain loading data of all TND membranes, Figure S10: Stress-strain loading data of all TNR membranes, Figure S11: Stress-strain loading data of all TNP membranes, Table S6: Summary Table.
