# Supplementary material for: Elasticity of Cross-Linked Titania Nanocrystal Assemblies Probed by AFM-Bulge Tests
Source: Nanomaterials (Basel). 2019 Aug 29;9(9):1230. doi: 10.3390/nano9091230 (PMC6780250; doi:10.3390/nano9091230)
Supplement: Supplementary file 1 [file nanomaterials-09-01230-s001.pdf]

Supplementary Materials

Elasticity of Cross-linked Titania Nanocrystal Assemblies Probed by AFM-bulge Tests

Andreas Hensel [1, †], Clemens J. Schröter [1, †], Hendrik Schlicke [1,2], Norbert Schulz [3], Svenja Riekeberg [3,4], Hoc Khiem Trieu [3], Andreas Stierle [5,6], Heshmat Noei [5], Horst Weller [1] and Tobias Vossmeyer [1,*]

[1] Institute of Physical Chemistry, University of Hamburg, Grindelallee 117, D-20146 Hamburg, Germany

[2] Present Address: Fraunhofer Center for Applied Nanotechnology CAN, Grindelallee 117, D-20146 Hamburg, Germany

[3] Institute of Microsystems Technology, Hamburg University of Technology, Eißendorfer Straße 42, D-21073 Hamburg, Germany

[4] Present Address: Thermo Fisher Scientific (Bremen) GmbH, Hanna-Kunath-Straße 11, D-28199 Bremen, Germany

[5] Deutsches Elektronen-Synchrotron (DESY), Notkestraße 85, D-22607 Hamburg, Germany

[6] University of Hamburg, Physics Department, Luruper Chaussee 149, D-22761 Hamburg, Germany

* Correspondence: tobias.vossmeyer@chemie.uni-hamburg.de, Fax: +49 40 42838 3452; Tel: +49 40 42838 7069

† These authors contributed equally to this work.

Contents

S1	Silicon substrates with circular apertures	3-4
S2	Elemental analyses	5
S3	Thermogravimetric analyses data	6
S4	Coverage of titania nanocrystal surfaces	7-8
S5	X-ray powder diffraction data	9-10
S6	Summary of titania nanocrystal properties	11
S7	Atomic force microscopy data	12
S8	X-ray photoelectron spectroscopy data	13-14
S9	Stress-strain data	15-18
S10	Micromechanical data	19
S11	References	20

S1 Silicon substrates with circular apertures

For the bulge tests silicon substrates with circular apertures were produced via deep reactive ion etching (DRIE). To fabricate defined circular apertures a photoresist is deposited via spin-coating onto a silicon substrate and structured by photolithography (Figure S1 A). Afterwards the photoresist is utilized as a mask for the DRIE process.

The DRIE process consists of two alternating steps. In the first step the silicon wafer is etched almost isotropically using a sulfur hexafluoride (SF_6) plasma (Figure S1 B). While chemical dry etching with highly reactive SF_6 plasma results in an isotropic etch behavior a bias voltage is applied to accelerate ions from the plasma in the direction of the wafer. These ions attack the wafer vertically and remove material by physical etching everywhere they hit the surface. The second step is a passivation step in which all surfaces are coated with a thin fluoropolymer layer. This layer is resistant to the chemical etchants which are used in the first step (Figure S1 C).

In the beginning of the next etching step the ions from the plasma attack the polymer at the bottom of the structure and open a section of silicon which can be etched again by the chemical dry etching. The walls of the structure are not attacked by the ions and therefore still passivated against the etchant (Figure S1 D).

By alternating these two steps a nearly vertical etch profile is achieved (Figure S1 E). After several hundred iterations the desired structure is finished. Figure S2 shows an SEM image of an aperture produced in a silicon substrate by this process.

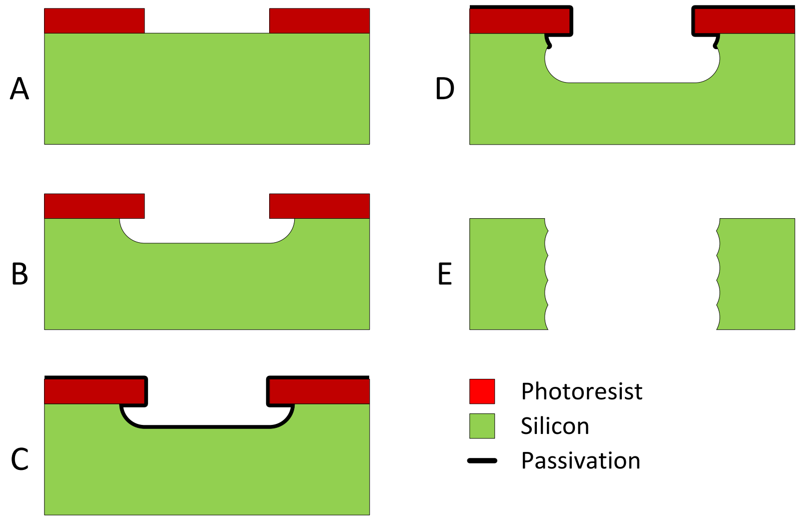

Figure S1. ***Deep reactive ion etching process*** used to prepare silicon substrates with circular apertures: Photolithographically patterned resist on silicon substrate (A), first etching step (B), first passivation step (C), second etching step (D), final etch profile (E).

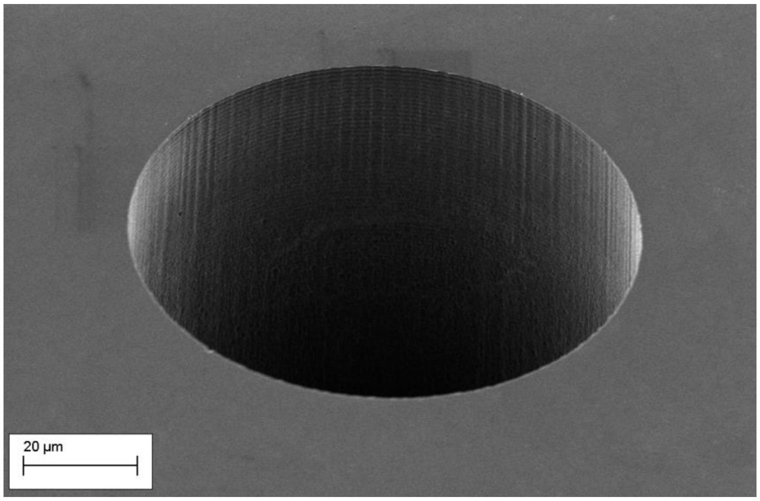

Figure S2. ***Scanning electron microscopy (SEM)-Image of a circular aperture*** (diameter: ~90 µm) etched into a silicon substrate via deep reactive ion etching.

S2 Elemental analyses

The three titania nanocrystal (TNC) samples that were used for the layer-by-layer deposition of TNC films were characterized by elemental analyses.

Table S1. ***Elemental analyses for carbon (C), hydrogen (H) and nitrogen (N)*** of as prepared titania nanodots (TNDs), nanorods (TNRs), and nanoplates (TNPs). The samples were dried at 60 °C and investigated via combustion analysis using a EuroEA Elemental Analyzer from (EuroVektro/Hekatech). The amount of oxygen could not be determined as titanium affects the measurement. Results are presented as % (w/w).

	**C**	**H**	**N**
TND	33.94 %	6.13 %	1.93 %
TNR	30.67 %	5.06 %	1.13 %
TNP	39.18 %	6.75 %	1.85 %

Table S2. ***Elemental analyses of TNDs, TNRs, and TNPs***, presented as molar fractions normalized to the amount of nitrogen.

	**C/N**	**H/N**	**N/N**
TND	20.51	44.14	1
TNR	31.65	62.17	1
TNP	24.76	50.81	1

Table S3. ***Molar ratios of the two ligands*** expected in the titania nanocrystal (TNC) samples: oleylamine (OLAM) and diethylene glycol (DEG) for TNDs; OLAM and oleic acid (OLAC) for TNRs and TNPs. The values presented were calculated from the data of Table S2: 18 carbon atoms were attributed to one nitrogen atom to account for the OLAM ($C_{18}H_{37}N$) ligand. For the TND sample the remaining carbon atoms were assumed to be part of residual DEG ($C_4H_{10}O_3$). For the TNR and TNP samples the remaining carbon atoms were assumed to be part of OLAC ($C_{18}H_{34}O_2$).

	**OLAM/OLAC**	**OLAM/DEG**
TND		1.60/1
TNR	1.32/1	
TNP	2.66/1	

S3 Thermogravimetric analyses data

The three TNC samples that were used for the layer-by-layer deposition of TNC films were characterized by thermogravimetric analysis (TGA).

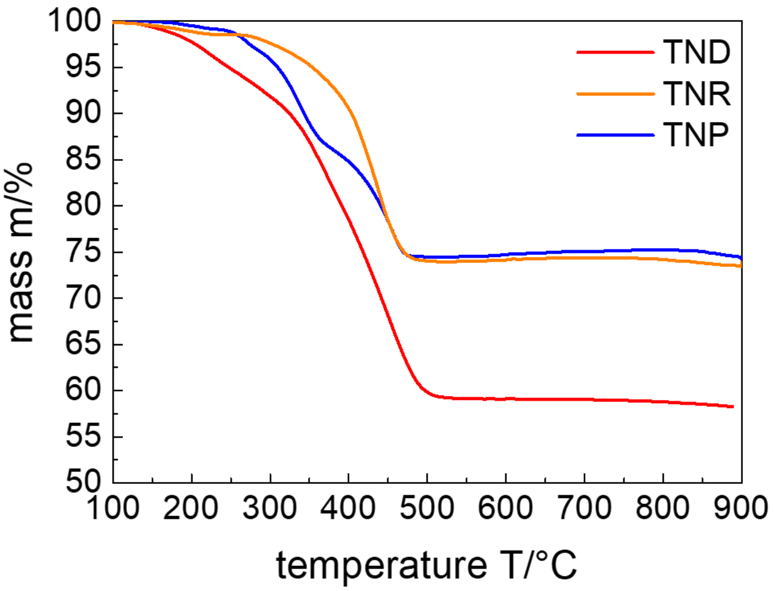

Figure S3. ***Thermogravimetric analyses*** of TND (red), TNR (orange) and TNP (blue) particles as used for spin-coating.

S4 Coverage of titania nanocrystal surfaces

To estimate the surface area of the TNCs we assumed the following geometries: A sphere with a diameter of 5 nm was assumed in case of the titania nanodots (TNDs). A cylinder with circular base area having a diameter of 3.4 nm and a length of 29.0 nm was assumed for the titania nanorods (TNRs). A square plate with an edge length of 33.6 nm and a thickness of 6.0 nm was assumed in the case of the titania nanoplates (TNPs).

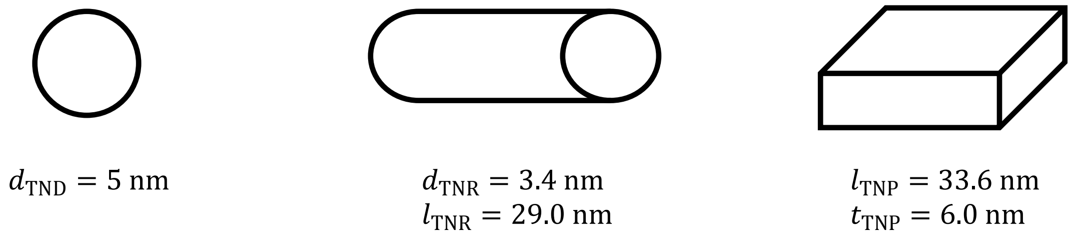

Figure S4. ***Geometries and dimensions*** used to estimate the surface area and volume of TNDs, TNRs, and TNPs.

For oleylamine (OLAM) and oleic acid (OLAC) ligands a footprint of $0.2\ \mathrm{nm}^2$ was assumed, according to literature for ammonia and acetic acid adsorbed onto titania anatase surfaces [1,2]. Dividing the TNC surface area by this footprint returns the amount of adsorbed ligands: 6.52×10^{-22} mol for a single TND, 2.72×10^{-21} mol for a single TNR, and 2.54×10^{-20} mol for a single TNP.

Elemental analyses of the TNR and TNP samples indicate that OLAM/OLAC is present at a molar ratio of 1.32/1 and 2.66/1, respectively (see Table S3). Thus, for TNRs and TNPs it was assumed that both ligands are bound at these molar ratios to the nanocrystal surface. With this assumption an average molecular mass of the ligands attached to the nanocrystals surface was calculated. For the TNDs it was assumed that only the OLAM ligands (of the OLAM/diethylene glycol (DEG) mixture, see Table S3) are bound to the nanocrystal surface. The respective molecular masses of the ligands were then used to calculate the total mass of ligands corresponding to one monolayer coverage of the TNCs.

Further, the average inorganic mass of the nanocrystals (without the ligands attached) was estimated by using the volumes of the idealized nanocrystal geometries (Figure S4) and the density of anatase (3.9 g/cm^3) or brookite (4.1 g/cm^3) for the TNDs and TNPs, or TNRs, respectively.

Using the calculated mass of the ligand monolayer covering the TNCs, and the mass of the inorganic titania nanocrystals, the organic mass fractions corresponding to one monolayer coverage was estimated as 40.8 % for the TNRs, 20.7 % for the TNPs, and 40.6 % for the TNDs.

The TGA data shown in Figure S3 reveal organic mass fractions of ~25 % for, both, TNRs and TNPs. Thus, it can be concluded that the TNRs were covered by roughly half a monolayer of ligands, whereas the TNPs were covered by roughly one monolayer of ligands.

The TGA data for the TNDs shown in Figure S3 reveals a total organic mass fraction ~40 %. However, in this case we consider only the OLAM ligand. Thus, taking into account the results of the elemental analysis for the TNDs (Table S3) and the molecular masses of OLAM and DEG, only a ~30 % mass loss (instead of the TGA measured 40 % mass loss) is attributed to OLAM, which still amounts to an OLAM ligand coverage of ~0.8 monolayers. Thus, the TNDs are also covered by roughly one ligand monolayer.

In summary, the estimations outlined above suggest that the TNCs, which were used for the layer-by-layer deposition of TNC films, were covered by roughly 0.5 - 1 monolayer of corresponding ligands.

S5 X-ray powder diffraction data

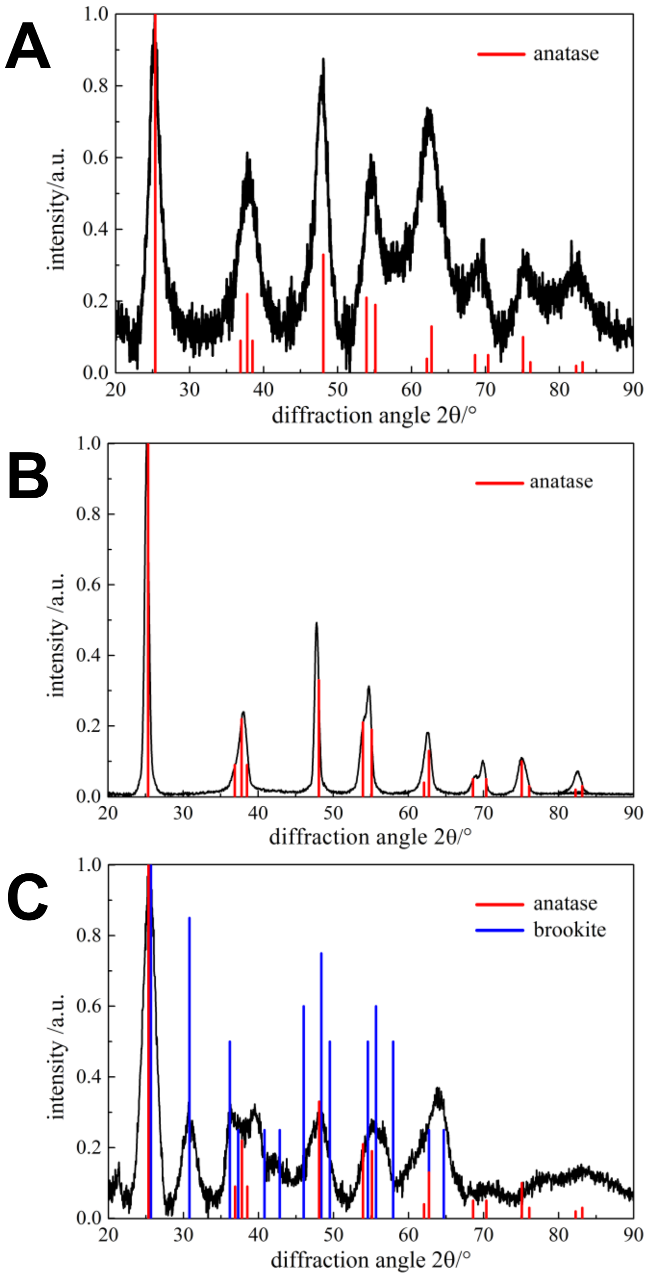

Figure S5. ***X-ray powder diffraction (XRD) data*** of TNDs (A), TNPs (B), and TNRs (C) with reference data for anatase (X'Pert Database: 00-004-0477; red) and brookite (X'Pert Database:00-015-0875; blue).

Figure S5 A presents the X-ray powder diffraction (XRD) data for TNDs. The crystalline domain size $\boldsymbol{L}$ was calculated using the Scherrer equation (see below). For this purpose, the full width at half maximum (FWHM) $\boldsymbol{w}$ was determined by fitting lorentzians to the (101) and (200) reflections. To account for instrumental broadening $\boldsymbol{b} = 0.06°$ was subtracted from the measured FWHM. The obtained $\boldsymbol{w}$ values, the $2\boldsymbol{\theta_0}$ center positions of respective reflections, as well as the calculated values for the crystalline domain sizes $\boldsymbol{L}$, are presented in Table S4. Assuming spherical crystallites, we used the shape factor $\boldsymbol{K}$ = 1.0747 [3]. The wavelength $\boldsymbol{\lambda}$ of our XRD instrument was 0.154 nm (Cu K_α).

$$L = \frac{K\,\lambda}{(w-b)\cdot\,\cos\theta_0}\frac{180°}{\pi}$$

Table S4. ***Evaluation of XRD data*:** Full with at half maximum $\boldsymbol{w}$, peak center position $\boldsymbol{2\theta_0}$ of the (101) and (200) reflections, and crystalline domaine sizes $\boldsymbol{L}$ of TNDs calculated using the Scherrer equation.

**peak**	$\boldsymbol{w}$ /°	$\boldsymbol{2\theta_0}$/°	$\boldsymbol{L}$/nm
(101)	1.67	25.27	6.04
(200)	1.93	47.82	5.55

S6 Summary of titania nanocrystal properties

Table S5. ***Properties of nanocrystal samples used for film preparation.*** Nanoparticle shape (by TEM), crystallite phase (by XRD), crystallite size (by TEM), organic mass fraction (by TGA), and calculated coverage by ligands as number of monolayers.

Sample	Shape	Crystallite Phase	Crystallite Size / nm	Organic Mass Fraction / %	Ligand Monolayers
TND	dots	anatase	diameter: 5	40	0.8
TNR	rods	brookite (+anatase)	diameter: 3.4<br>length: 29.0	25	0.6
TNP	plates	anatase	thickness: 6<br>edge length: 33.6	25	1.2

S7 Atomic force microscopy data

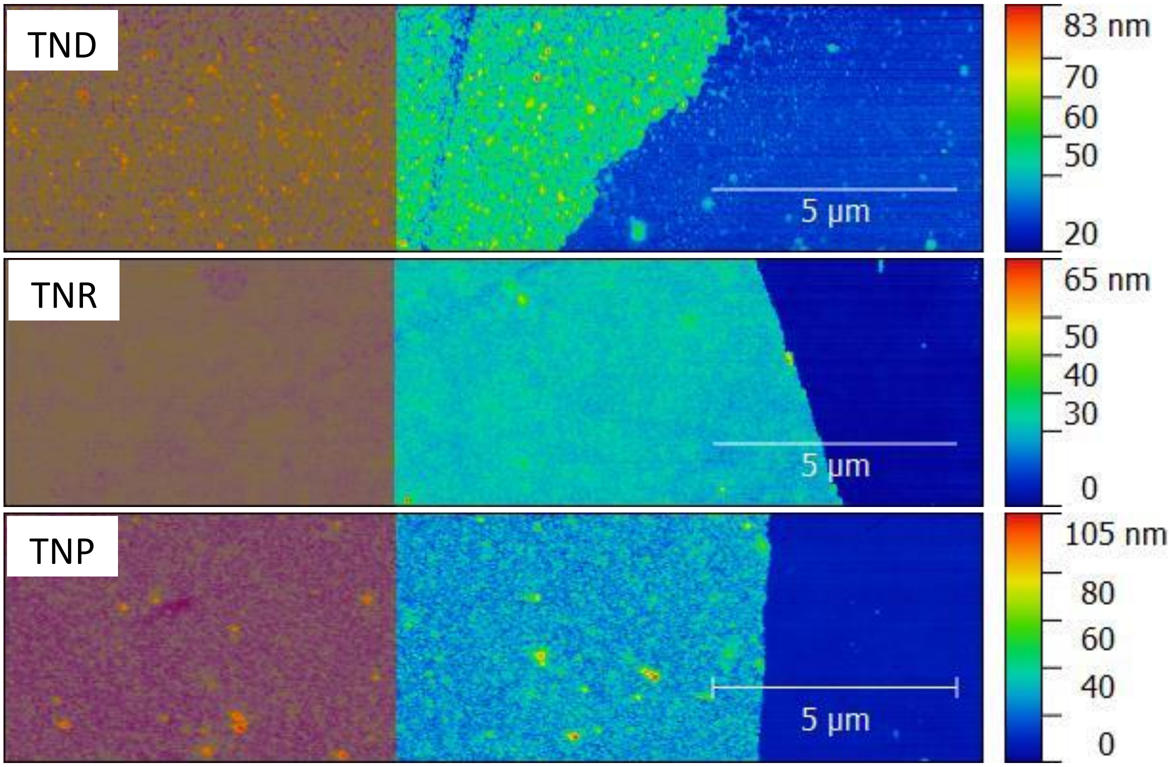

Figure S6. ***Atomic force microscopy (AFM) images*** of TND (5 deposition cycles), TNR (4 deposition cycles) and TNP (3 deposition cycles) films. The films were transferred onto silicon substrates for AFM measurements.

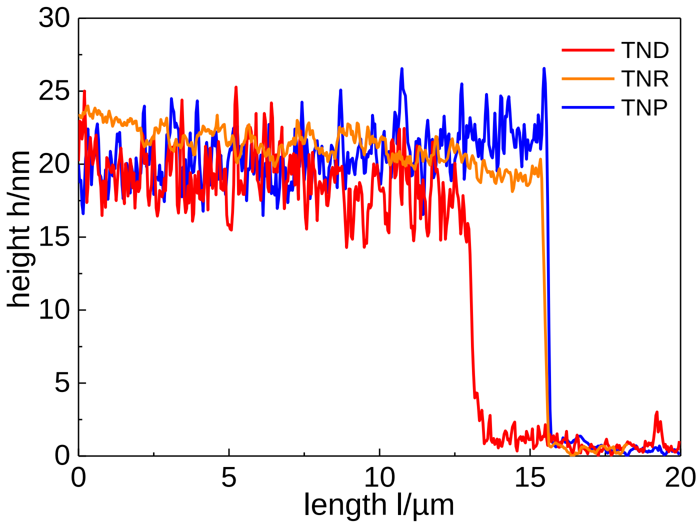

Figure S7. ***Height profiles*** extracted from the AFM images of Figure S6 for TND, TNR, and TNP films.

S8 X-ray photoelectron spectroscopy data

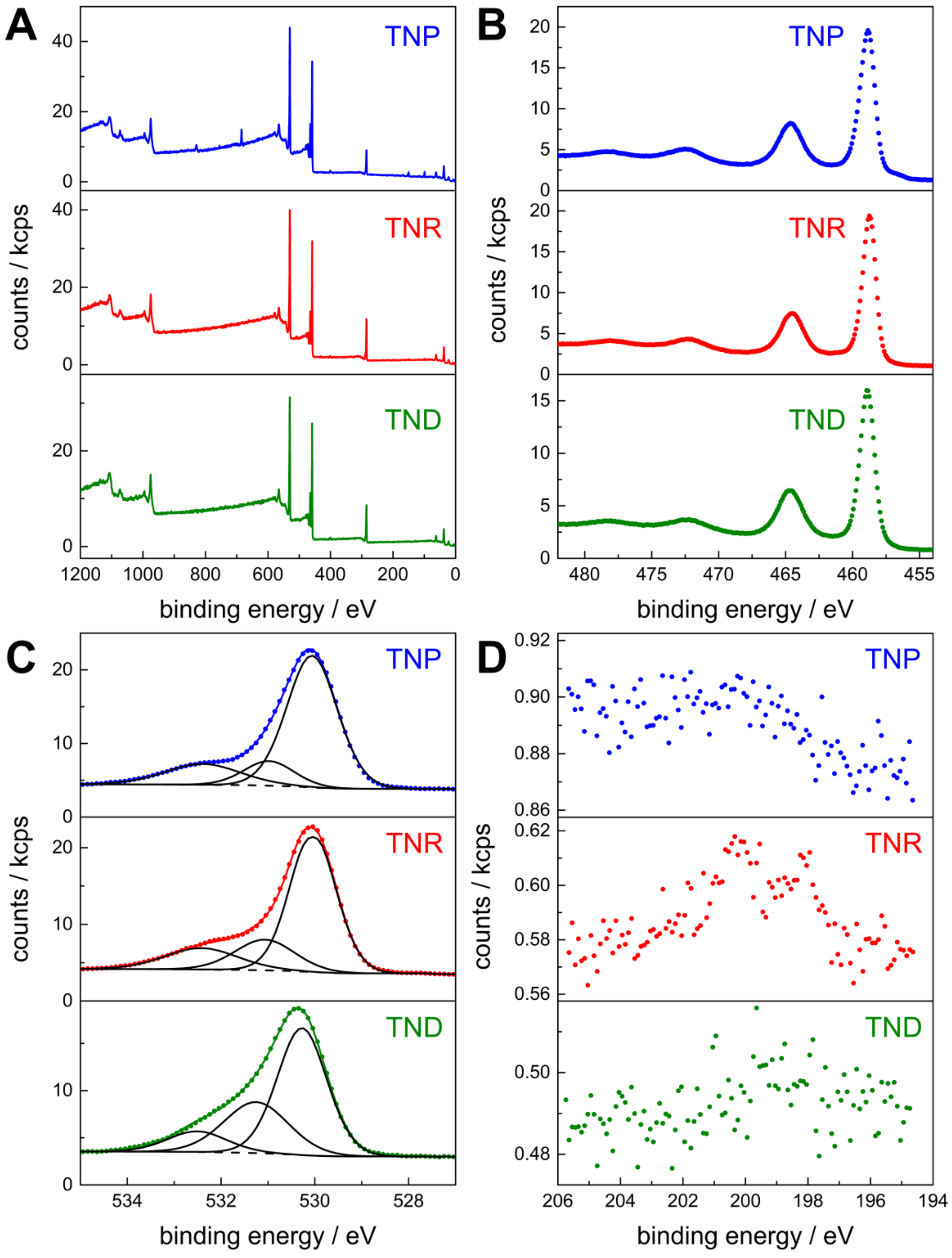

Figure S8. ***X-ray photoelectron spectroscopy (XPS) Data of TNC films***. Figure part (A) shows the survey spectra of TNP, TNR, and TND films. Figure part (B) shows the Ti 2p energy region. Figure part (C) shows the O 1s signals. The peaks at binding energies around 532.4 and 531.0 eV are attributed to carboxylic groups, while the peak at around 530.5 eV is attributed to the Ti-O species. Figure part (D) shows the Cl 2p energy region.

The strong X-ray photoelectron spectroscopy (XPS) signals assigned to carboxy carbon and carboxy oxygen clearly indicate the deposition of the 1,12-dodecanedioic acid (12DAC) cross-linker during layer-by-layer film preparation. As an estimate, we compare the XPS signals at 288.6 eV (assigned to carboxylic carbon, see Figure 4A of the main document) to the total XPS carbon signal. This comparison returns carboxylic carbon/total carbon ratios of 10.4 % for the TNR film, 9.5 % for the TNP film, and 9.4 % for the TND film.

Table S3 (see above) presents the OLAM/OLAC ratios for the TNR and TNP particle samples used for film preparation. These ratios were deduced from elemental analyses and correspond to carboxylic carbon/total carbon ratios of 2.4 % and 1.5 % for the TNR and TNP particles, respectively. (Note: there was no carboxylic acid involved in the preparation of the TND particles. Therefore, we assume that the carboxylic carbon/total carbon was zero for the TND particles.)

Thus, comparing the relative amounts of carboxylic carbon deduced from elemental analysis of the particle samples (that were used for film preparation) to that determined from the XPS signals of the TNC films, clearly reveals a significant increase of carboxylic carbon after film assembly.

S9 Stress-strain data

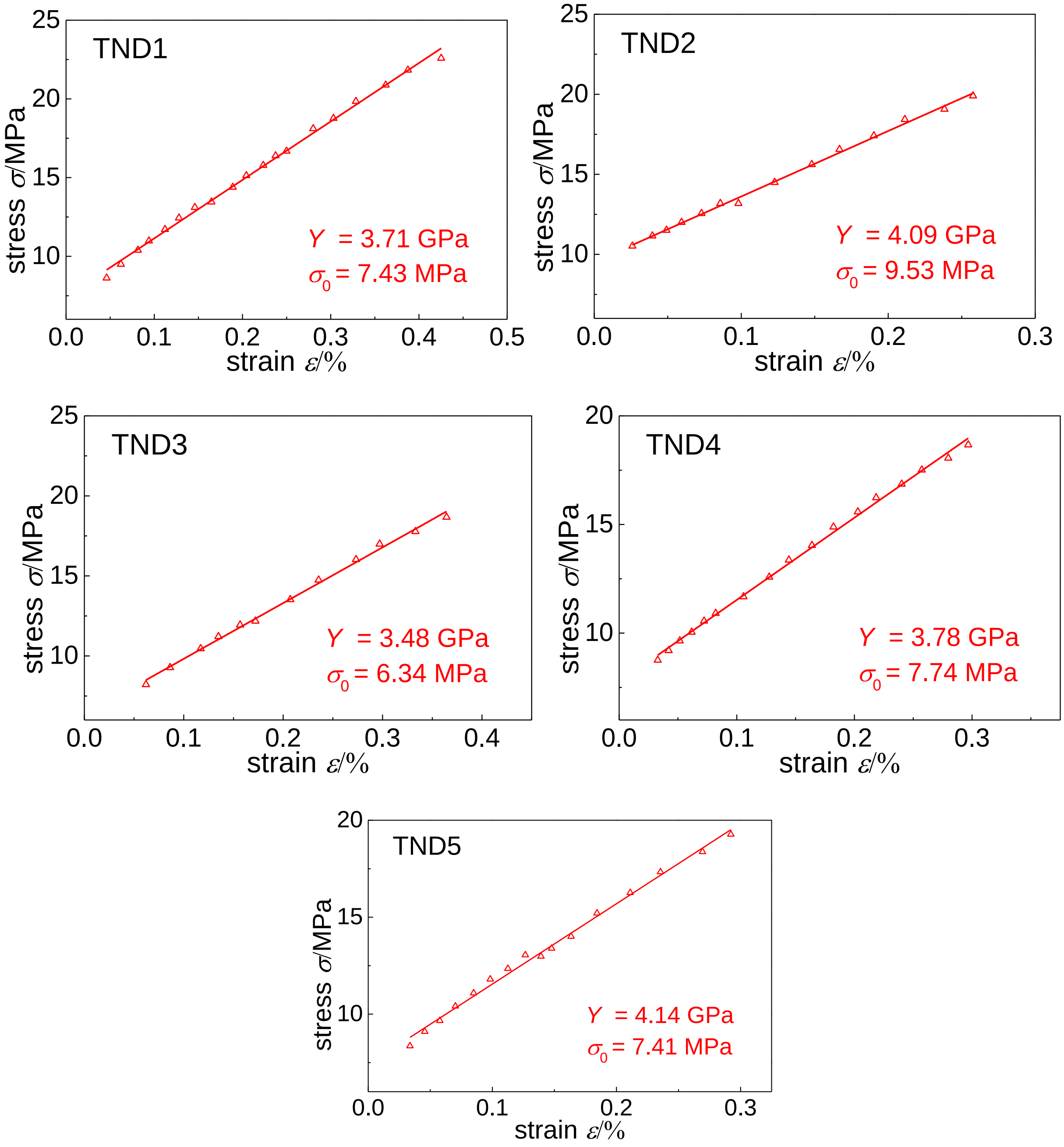

Figure S9. ***Stress-strain loading data of all TND membranes*** studied. The biaxial modulus ***Y*** and pre-stress $\boldsymbol{\sigma_0}$ were extracted as the slope and the ordinate intercept of the linear fit function. Additional information to each membrane can be found in Summary Table S5.

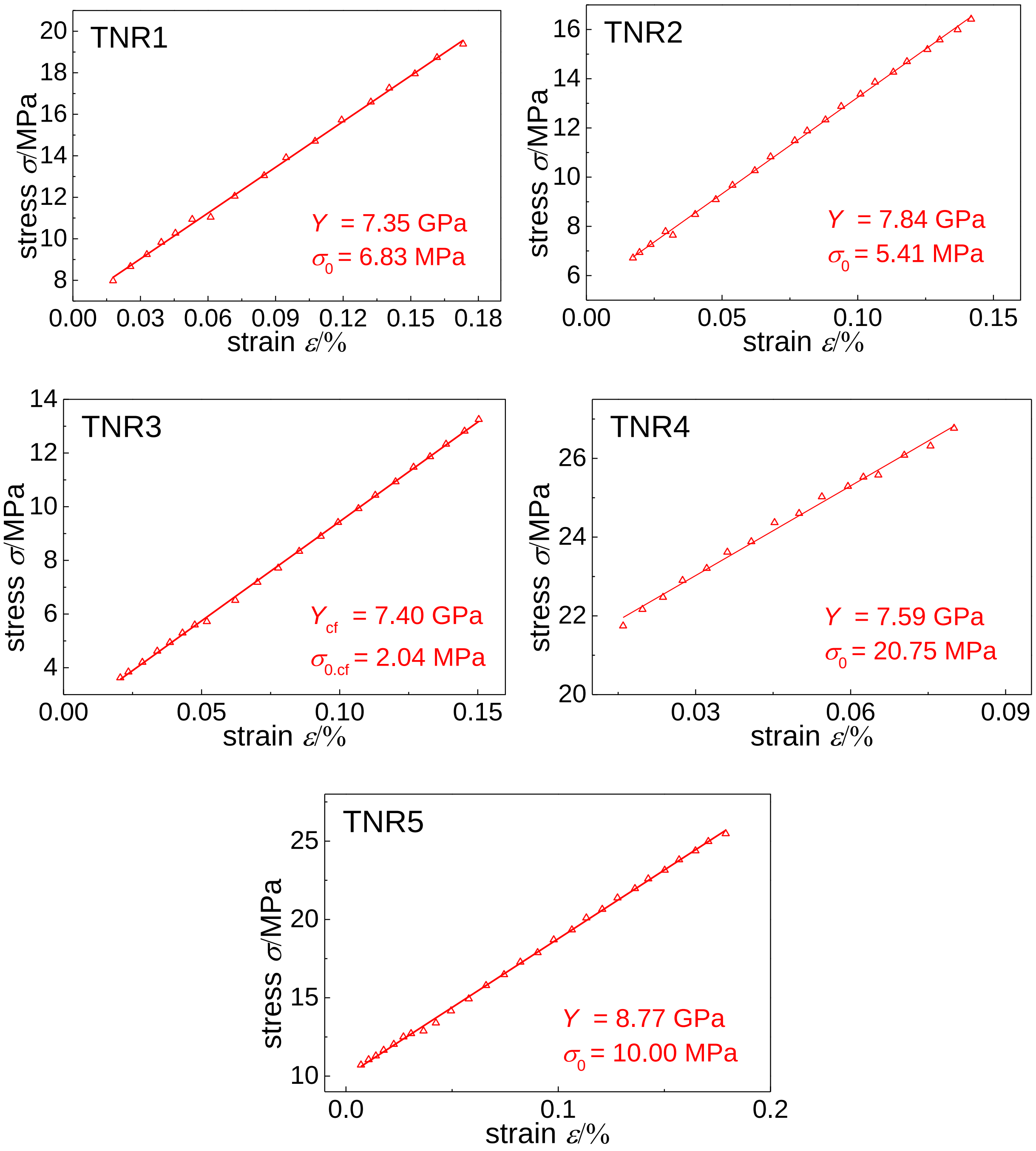

Figure S10. ***Stress-strain loading data of all TNR membranes*** studied. The biaxial modulus $\boldsymbol{Y}$ and pre-stress $\boldsymbol{\sigma_0}$ were extracted as the slope and the ordinate intercept of the linear fit function. Additional information to each membrane can be found in Summary Table S5.

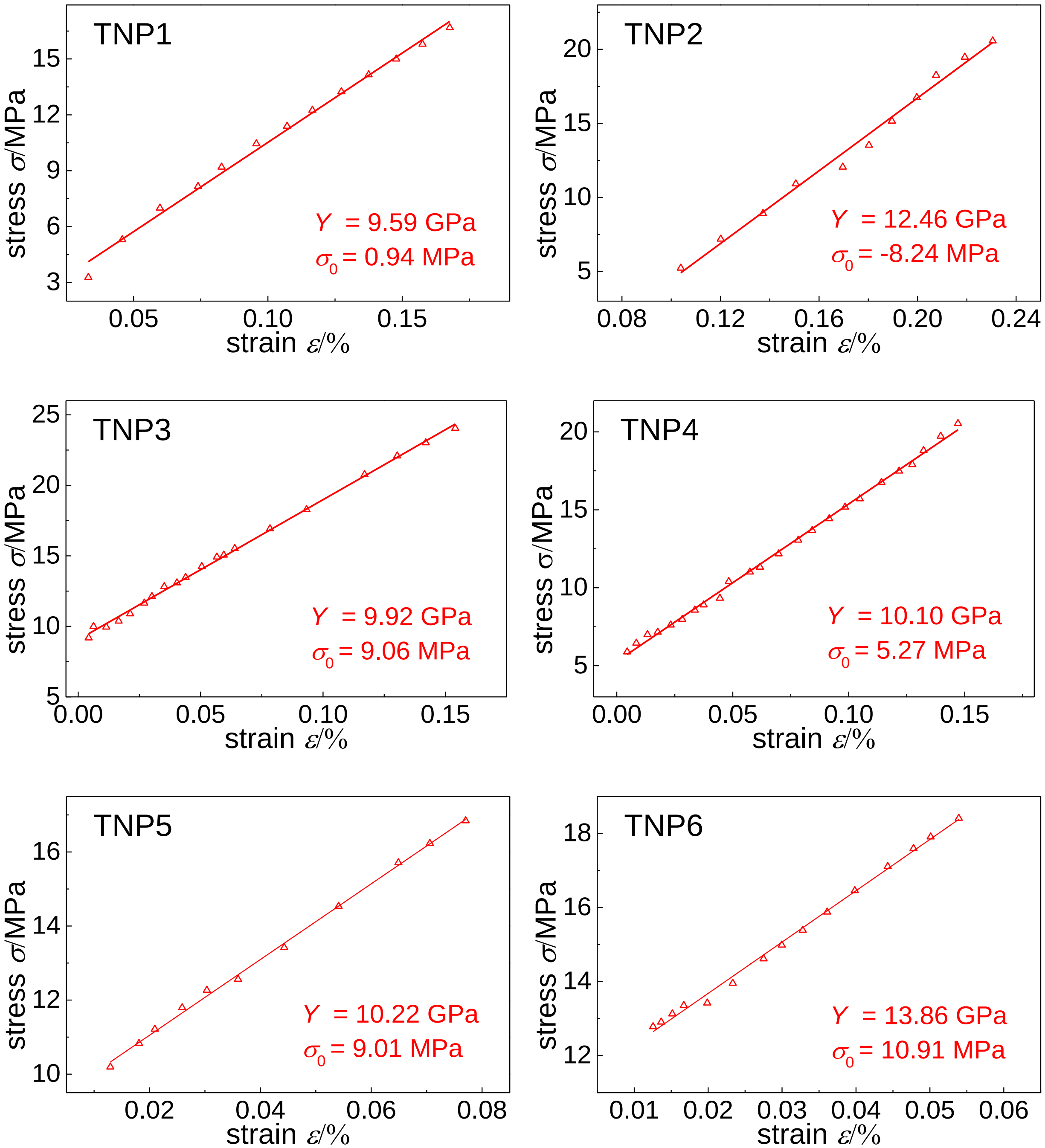
TNP1
Y = 9.59 GPa
σ0 = 0.94 MPa
stress σ/MPa
strain ε/%
3 6 9 12 15
0.05 0.10 0.15
TNP2
Y = 12.46 GPa
σ0 = -8.24 MPa
stress σ/MPa
strain ε/%
5 10 15 20
0.08 0.12 0.16 0.20 0.24
TNP3
Y = 9.92 GPa
σ0 = 9.06 MPa
stress σ/MPa
strain ε/%
5 10 15 20 25
0.00 0.05 0.10 0.15
TNP4
Y = 10.10 GPa
σ0 = 5.27 MPa
stress σ/MPa
strain ε/%
5 10 15 20
0.00 0.05 0.10 0.15
TNP5
Y = 10.22 GPa
σ0 = 9.01 MPa
stress σ/MPa
strain ε/%
10 12 14 16
0.02 0.04 0.06 0.08
TNP6
Y = 13.86 GPa
σ0 = 10.91 MPa
stress σ/MPa
strain ε/%
12 14 16 18
0.01 0.02 0.03 0.04 0.05 0.06

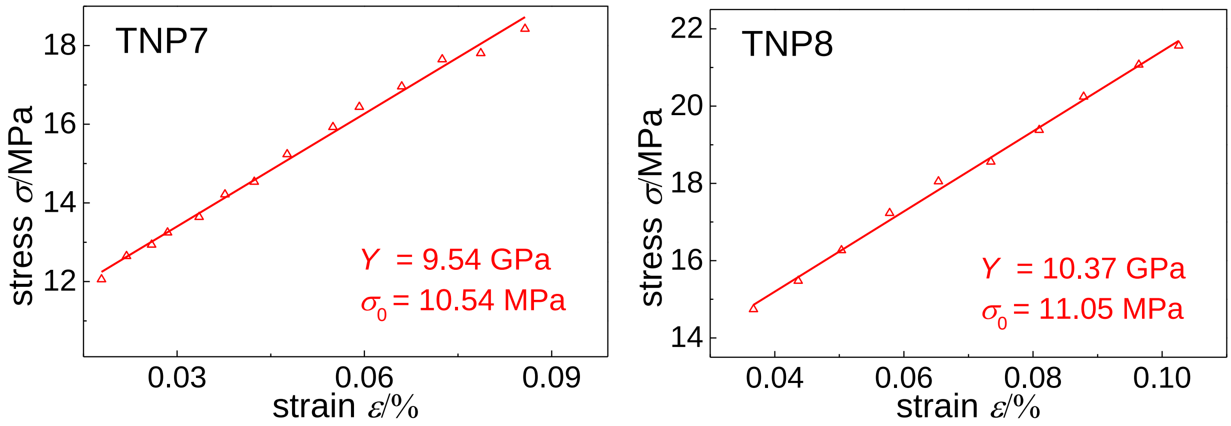

Figure S11. ***Stress-strain loading data of all TNP membranes*** studied. The biaxial modulus $\boldsymbol{Y}$ and pre-stress $\boldsymbol{\sigma_0}$ were extracted as the slope and the ordinate intercept of the linear fit function. Additional information to each membrane can be found in Summary Table S5.

S10 Micromechanical data

Table S6. ***Summary Table*****:** Geometric parameters (membrane thickness, aperture diameter), maximum stress applied during the AFM bulge test, biaxial modulus $\boldsymbol{Y}$, pre-stress $\boldsymbol{\sigma_0}$, and Young's modulus $\boldsymbol{E}$, of all membranes studied.

Membrane	Thickness t / nm	Aperture Size $2a$ / µm	Max Stress / MPa	Biaxial Modulus Y / GPa	Pre-Stress σ_0 / MPa	Elastic Modulus E / GPa
TND1	21.32	107.88	22.58	3.71	7.43	2.49
TND2	21.32	87.06	19.91	4.09	9.53	2.74
TND3	21.32	105.84	18.67	3.48	6.34	2.33
TND4	20.16	48.00	18.98	3.78	7.74	2.53
TND5	20.16	86.25	21.11	4.14	7.41	2.77
**Average**				**3.84±0.28**	**7.69±1.16**	**2.57±0.18**
TNR1	34.05	88.98	19.38	7.35	6.83	4.92
TNR2	37.25	51.06	16.42	7.84	5.41	5.25
TNR3	37.25	90.60	13.83	7.40	2.04	4.96
TNR4	39.33	66.03	26.77	7.59	20.75	5.09
TNR5	33.18	111.48	25.48	8.77	10.00	5.88
**Average**				**7.79±0.58**	**9.01±7.16**	**5.22±0.39**
TNP1	37.02	31.59	16.68	9.59	0.94	6.43
TNP2	33.02	88.17	20.57	12.46	-8.24	8.35
TNP3	33.02	86.63	24.04	9.92	9.06	6.65
TNP4	36.72	67.30	20.54	10.10	5.27	6.77
TNP5	37.02	84.26	25.62	10.22	9.01	6.85
TNP6	41.67	95.93	18.41	13.86	10.91	9.29
TNP7	41.67	65.17	18.43	9.54	10.54	6.39
TNP8	41.67	90.75	21.56	10.37	11.05	6.95
**Average**				**10.76±1.56**	**6.07±6.74**	**7.21±1.04**

S11 References

1. Koust, S.; Adamsen, K. C.; Kolsbjerg, E. L.; Li, Z.; Hammer, B.; Wendt, S.; Lauritsen, J. V. NH_3 adsorption on anatase-TiO_2(101*). J. Chem. Phys*. **2018**, *148*, 124704 (5pp).
2. Grinter, D. C.; Nicotra, M.; Thornton, G. Acetic Acid Adsorption on Anatase TiO2 (101), *J. Phys. Chem. C*, **2012**, *116*, 11643-11651.
3. Langford, J. I.; Wilson, A. J. C. Scherrer after Sixty Years: A Survey and Some New Results in the Determination of Crystallite Size. *J. Appl. Cryst.* **1978**, *11*, 102-113.
